# A long-term survivor of recurrent esophagogastric junction adenocarcinoma treated with multidisciplinary therapy: a case report

**DOI:** 10.1186/s40792-020-0776-5

**Published:** 2020-01-09

**Authors:** Takako Tanaka, Takaaki Arigami, Yoshikazu Uenosono, Shigehiro Yanagita, Daisuke Matsushita, Keishi Okubo, Takashi Kijima, Yasuto Uchikado, Yoshiaki Kita, Shinichiro Mori, Ken Sasaki, Itaru Omoto, Hiroshi Kurahara, Kosei Maemura, Sumiya Ishigami, Shoji Natsugoe

**Affiliations:** 1 Department of Digestive Surgery, Breast and Thyroid Surgery, Kagoshima University Graduate School of Medical and Dental Sciences, 35-1, Sakuragaoka 8 cho-me, Kagoshima City, Kagoshima 890-8520 Japan; 2 Department of Onco-biological Surgery, Kagoshima University Graduate School of Medical and Dental Sciences, 35-1, Sakuragaoka 8 cho-me, Kagoshima City, Kagoshima 890-8520 Japan

**Keywords:** Esophagogastric junction adenocarcinoma, Multimodal therapy, Recurrence, Surgical resection

## Abstract

**Background:**

Patients with esophagogastric junction cancer are increasing in Western and Eastern countries. Conversely, the clinical significance of surgical resection remains controversial in these patients. We report a long-term survivor of recurrent esophagogastric junction adenocarcinoma who underwent constructive multimodal therapy, including surgical resection.

**Case presentation:**

A 51-year-old man underwent total gastrectomy for esophagogastric junction adenocarcinoma in 2009. In June 2010, computed tomography (CT) indicated a lung nodule and we partially resected the right lower lung. It was pathologically diagnosed as distant metastasis from esophagogastric junction cancer. After lung resection, he received adjuvant chemotherapy with S-1 for 1 year. In September 2014, CT demonstrated a swelling of the upper mediastinal lymph node with abnormal uptake on fluorine-18 fluorodeoxyglucose positron emission tomography. We performed an ultrasonography-guided needle biopsy, and he was diagnosed with lymph nodal recurrence of esophagogastric junction adenocarcinoma by pathological examination and was subsequently treated with capecitabine plus cisplatin plus trastuzumab. Since CT showed a reduction in the metastatic upper mediastinal lymph node after chemotherapy, he underwent upper mediastinal lymphadenectomy in April 2015. Following surgery, we provided radiation therapy to the upper mediastinum and chemotherapy with S-1. At the last report, the patient was alive for 8 years and 3 months since the first surgery.

**Conclusions:**

This case report shows the clinical benefit of constructive multimodal therapy for recurrent esophagogastric junction adenocarcinoma.

## Background

The incidence of adenocarcinoma of the esophagogastric junction is increasing in Western and Eastern countries. However, the proposed strategies for the treatment in these patients are diverse, and there is no established consensus on the optimal treatment [1]. We report a long-term survivor with recurrent esophagogastric junction adenocarcinoma who underwent constructive multimodal therapy, including surgical resection.

## Case presentation

**Fig. 1:**
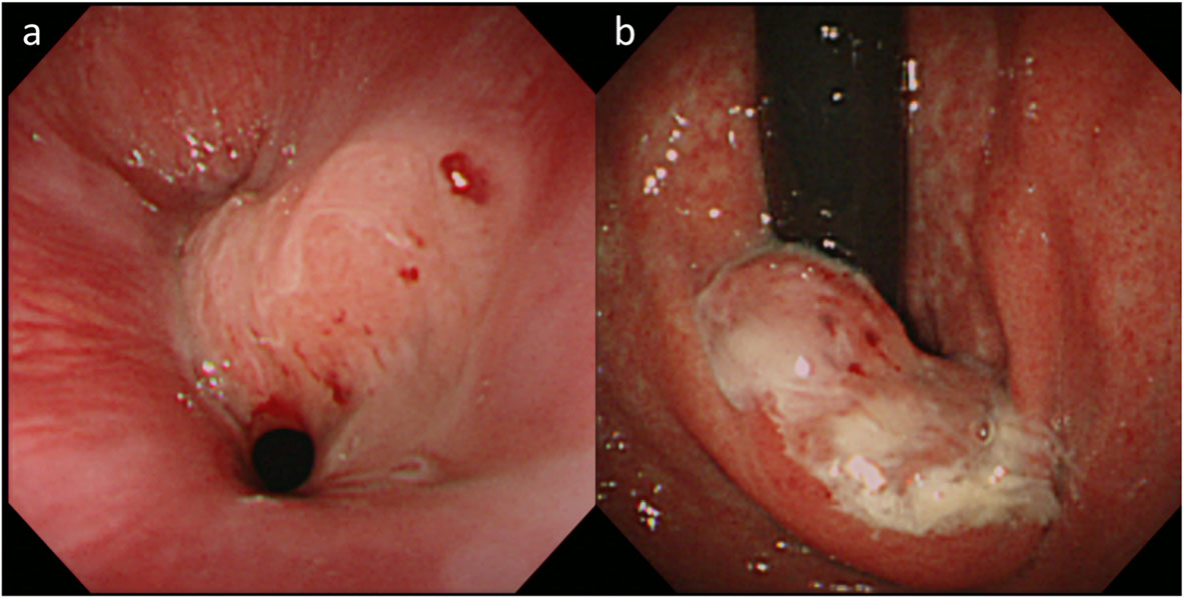
Esophagogastroduodenoscopy revealed a type 1 tumor in the esophagogastric junction (**a**, **b**)

**Fig. 2:**
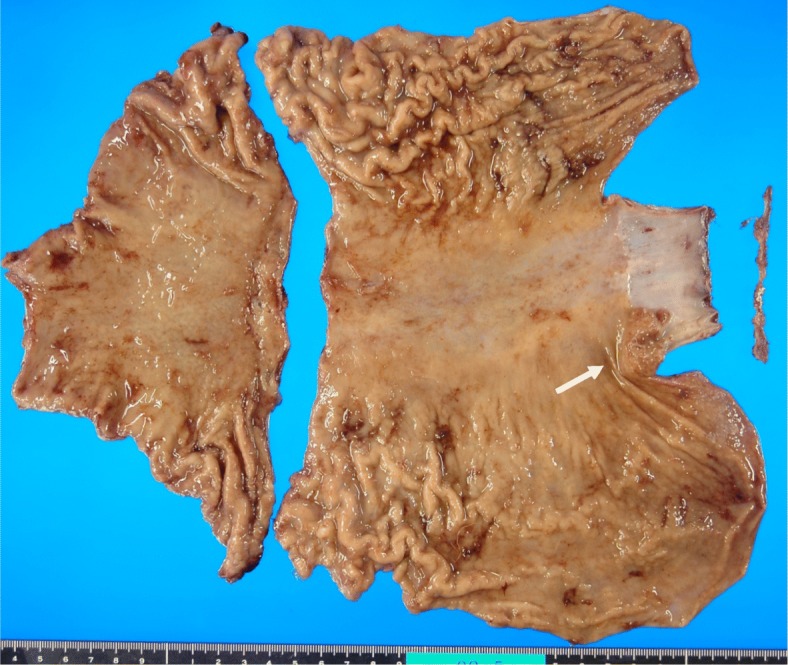
Macroscopic findings of the resected stomach. The tumor size was 25 × 17 mm (arrow)

**Fig. 3:**
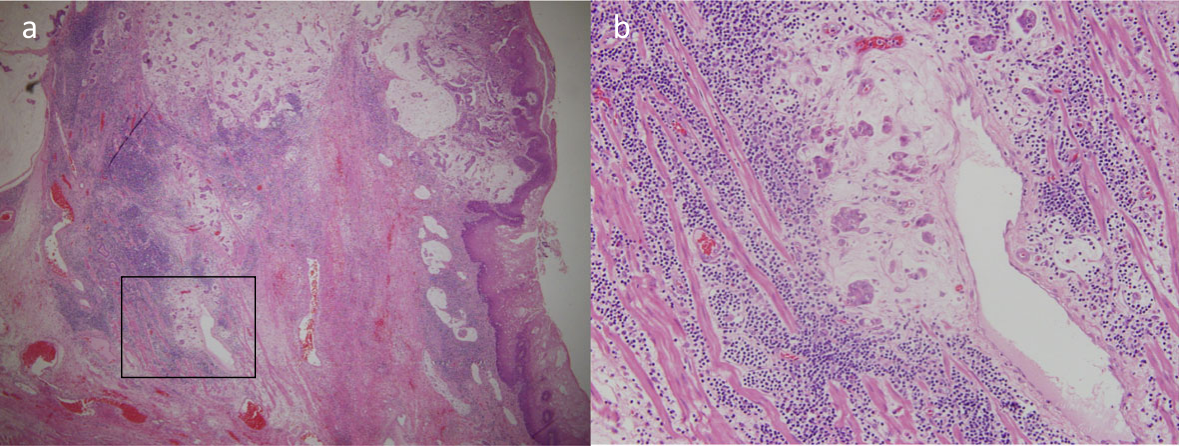
Histopathological findings of the primary tumor. Original magnification × 40 (**a**). Original magnification × 400 (**b**)

A 51-year-old man presented with an abnormal gastric shape on medical examination. Esophagogastroduodenoscopy revealed type 1 tumor of the esophagogastric junction (Fig. 1a, b). Pathological examination of the biopsied specimens showed a moderately tubular adenocarcinoma with mucinous adenocarcinoma. Abdominal computed tomography (CT) did not indicate swelling of any lymph node or distant metastasis. On the basis of these clinical and pathological findings, we diagnosed the patient with Siewert type II esophagogastric junction adenocarcinoma (T2 N0 M0 stage IB). Although we initially performed proximal gastrectomy in January 2009, we soon converted to total gastrectomy with D2 lymphadenectomy due to the intrasurgical detection of a lymph node metastasis in station no. 2. The resected specimen showed tumor measuring 25 × 17 mm (Fig. 2), and the tumor was identified as mucinous adenocarcinoma on the basis of histological classification (Fig. 3a, b). According to the pathological examination of resected specimens, his tumor was T1b N1 M0 stage IB and HER2 status was negative. He was followed up every 2 to 3 months without adjuvant chemotherapy by regular clinical diagnostic examinations, such as tumor marker studies (CEA and CA19-9) and computed tomography.

In June 2009, CT showed an isolated small nodule measuring 3 mm in the right lower lobe of the lung. Because the lesion exhibited normal uptake on fluorine-18 fluorodeoxyglucose positron emission tomography (FDG-PET), the patient was followed up with close observation only.

**Fig. 4:**
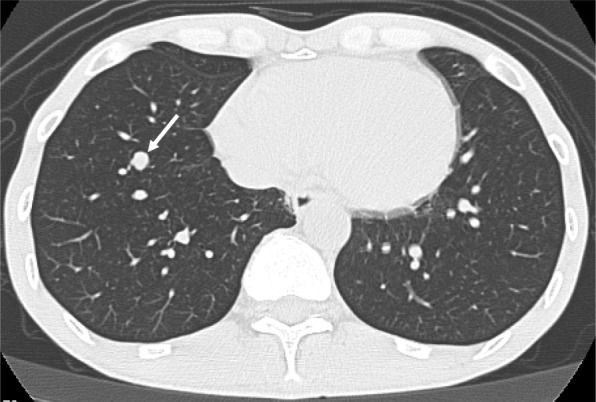
Computed tomography showed an enlarged nodule in the right lower lung lobe (arrow)

In June 2010, CT indicated that the lung nodule had increased to 8 mm in size (Fig. 4). We partially resected the right lower lung, including the nodule. Since pathological examination showed the resected lung tumor was a mucinous adenocarcinoma with positive expression of cytokeratin (CK) 7 and negative expression of CK 20, thyroid transcription factor-1, and apoprotein A, it was judged as a distant metastasis from the esophagogastric junction adenocarcinoma. HER2 status in lung metastatic lesion was not explored due to primary tumor with HER2 negative.

**Fig. 5:**
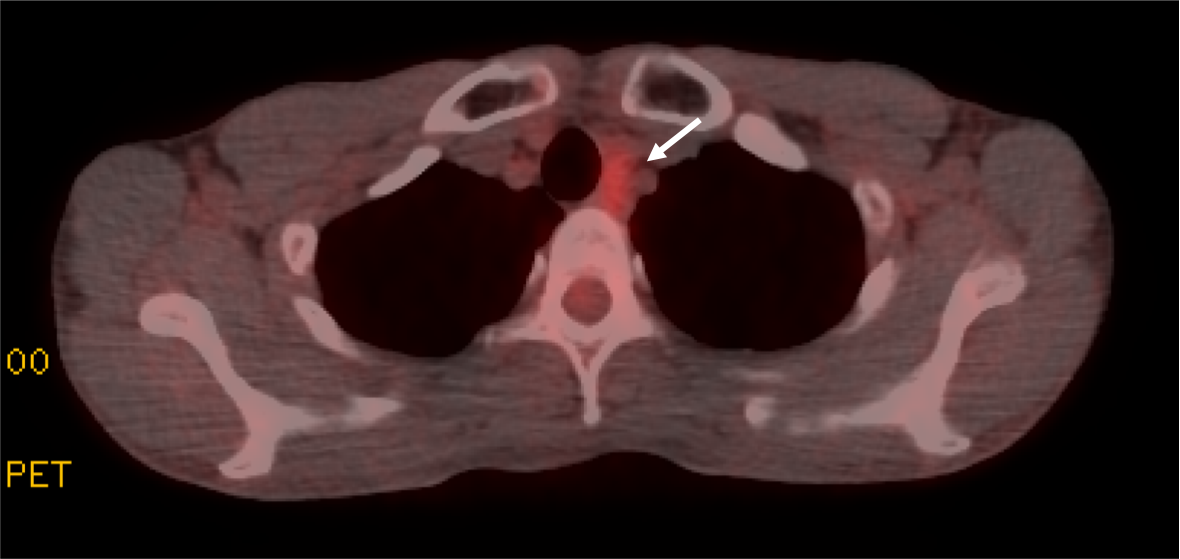
Fluorine-18 fluorodeoxyglucose positron emission tomography indicated abnormal uptake (arrow) within the upper mediastinal lymph node

After lung resection, he received adjuvant chemotherapy with S-1. The treatment schedule was the oral administration of S-1 (120 mg/body) for 4 weeks, followed by 2 weeks of rest, repeated every 6 weeks. This adjuvant chemotherapy with S-1 was continued for 1 year. Although a May 2013 CT showed swelling of the upper mediastinal lymph node, the maximum standardized uptake value (SUV_max_) in FDG-PET was 2.3. Consequently, he was followed up by observation. In September 2014, FDG-PET indicated that this node now demonstrated increased FDG uptake (SUV_max_, 2.8) (Fig. 5). The lymph node specimens obtained by ultrasonography-guided needle biopsy appeared to be mucinous adenocarcinoma, identical to this patient’s esophagogastric junction cancer. He was enrolled in a clinical trial and received five courses of trastuzumab in combination with capecitabine and cisplatin. The therapeutic schedule included the oral administration of capecitabine (3000 mg/body) for 2 weeks, followed by 1 week of rest, repeated every 3 weeks, and administration of both cisplatin (80 mg/m^2^) and trastuzumab (8 mg/kg in the first course and 6 mg/kg after second courses) on day 1.

Following chemotherapy, CT showed decreased size of the metastatic lymph node. In April 2015, he underwent left upper mediastinal lymphadenectomy by the left cervical approach. Mucinous adenocarcinoma was identified by pathological examination. Although we proposed chemoradiotherapy after surgery, he refused our suggestion. Accordingly, he underwent radiation therapy with a total of 50.4 Gy introduced into the upper mediastinal field. In September 2015, he was treated with the oral administration of S-1 (80 mg/body) for 2 weeks, followed by 2 weeks of rest, repeated every 4 weeks. Since he could not continue chemotherapy because of grade 2 neutropenia and grade 1 fatigue, we stopped chemotherapy after only two courses. At 8 years and 4 months after the initial surgery, this patient exhibited no signs of disease recurrence on follow-up examinations.

## Discussion

It is well known that the process of recurrence and the metastatic pathway are completely different from primary organs or histological types [2]. Most patients with pulmonary metastases from gastric cancer have carcinomatous lymphangiomatosis or carcinomatous pleuritic [3]. The incidence of surgically resectable solitary metastases is reportedly only 0.1% [4], and the clinical significance of surgical resection remains unclear in patients with pulmonary metastases from gastric cancer. Table 1 summarizes six reports, published from 2000 to 2012, on Japanese patients who underwent surgical resection for pulmonary metastases from gastric cancer [2, 5–9].

**Table 1:** Reported case of resected lung metastasis from gastric cancer in Japan

Year	Author	Age	Sex	Depth of invasion	Pathological stage	*N*	Histological type	Type of gastrectomy	Disease-free interval	Adjuvant chemotherapy	Number of pulmonary metastases	Metastatic lesion	Outcome after gastrectomy
2002	Inoue et al. [5]	68	M	sm	Stage Ia	N0	tub1	DG	8 years	Unknown	1	Lung	A (15 years)
2002	Tamura et al. [6]	67	M	ss	Stage IIA	N1	tub2	TG	5 years, 8 months	Unknown	1	Lung Brain	D (6 years, 2 months)
2002	Tamura et al. [6]	47	M	se	Stage IIIA	N1	tub2	TG	4 years, 1 month	Unknown	1	Lung Chest wall	D (5 years, 7 months)
2002	Tamura et al. [6]	73	M	se	Stage IIIA	N1	muc	TG	2 years	Unknown	1	Lung Liver	D (2 years, 7 months)
2002	Tamura et al. [6]	65	M	mp	Stage IB	N0	tub2	TG	7 months	Unknown	1	Lung Brain	D (1 year, 7 months)
2007	Sakaguchi et al. [7]	67	F	si	Stage IIIA	–	pap	TG	4 years, 1 month	5FU + CDDP	1	Lung	A (10 years, 5 months)
2007	Sakaguchi et al. [7]	75	M	mp	Stage IB	–	pap	DG	5 years	Unknown	1	Lung	A (7 years, 9 months)
2007	Sakaguchi et al. [7]	57	M	ss	Stage II	–	tub2	DG	4 years	Unknown	1	Lung	A (5 years, 9 months)
2007	Sakaguchi et al. [7]	75	M	sm	Stage IB	–	tub2	TG	4 years	Unknown	1	Lung	A (4 years, 8 months)
2007	Sakaguchi et al. [7]	57	F	ss	Stage IB	–	tub2	TG	1 years, 8 months	S-1	1	Lung	A (3 years, 5 months)
2007	Sakaguchi et al. [7]	76	F	ss	Stage II	–	tub1	TG	0 months	Unknown	1	Lung	D (4 years, 11 months)
2007	Sakaguchi et al. [7]	84	M	ss	Stage IB	–	tub1	TG	19 months	Unknown	1	Lung	D (1 year, 10 months)
2009	Nishioka et al. [8]	40	M	se	Stage IIIA	N1	pap	PG	4 years	S-1 + CPT11	2	Lung	A (7 years)
2012	Harada et al. [2]	55	M	se	Stage II	N0	tub2	TG	3 years	S-1	2	Lung Adrenal gland	A (6 years, 9 months)
2012	Shimoyama et al. [9]	55	M	si	Stage IV	N3	tub2	DG	7 years, 2 months	Unknown	1	Lung	A (8 years, 5 months)
2012	Shimoyama et al. [9]	77	M	ss	Stage II	N1	tub1	DG	5 years	Unknown	1	Lung	D (9 years, 8 months)
2012	Shimoyama et al. [9]	76	M	ss	Stage II	N1	tub2	TG	3 years, 7 months	Unknown	1	Lung Liver Brain	D (4 years, 11 months)
2012	Shimoyama et al. [9]	80	M	ss	Stage IB	0	tub2	TG	1 year, 6 months	Unknown	3	Lung	D (3 years, 8 months)
2019	Our case	51	M	sm	Stage IB	N1	muc	TG	1 years, 5 months	S-1, cape+CDDP+Trastuzumab	1	Lung	A (8 years, 4 months)

*sm* submucosa, *si* invasion of adjacent structures, *mp* muscularis propria, *se* serosa exposed, *ss* subserosa, *tub1* well-differentiated tubular adenocarcinoma, *pap* papillary adenocarcinoma, *tub2* moderately differentiated tubular adenocarcinoma, *muc* mucinous adenocarcinoma, *DG* distal gastrectomy, *TG* total gastrectomy, *PG* proximal gastrectomy, *5FU* fluorouracil, *CDDP* cisplatin, *cape* capecitabine, *CPT11* irinotecan, *A* alive, *D* dead

In the present case, tumor recurred within the lung and upper mediastinal lymph nodes metachronously. Interestingly, patients with synchronous metastasis of other organs in addition to pulmonary metastases tend to have poor prognosis (Table 1). Since our patient had a metachronous recurrence of pulmonary and lymph node metastases after gastrectomy, his survival may be prolonged.

Presently, chemotherapy is the standard treatment in patients with unresectable advanced or recurrent gastric cancers. Overall survival was significantly better among patients with unresectable advanced or recurrent gastric cancer who were randomized to receive chemotherapy, compared with those who received supportive care only [10]. The Gastric Cancer Treatment Guidelines, published by the Japanese Gastric Cancer Association in 2018, include recommended chemotherapies [11]. Our patient received a trastuzumab-containing regimen, and his metastatic lymph node shrank after five courses. Recent advances in anticancer agents, including novel molecular-targeted drugs such as trastuzumab, have helped improve prognosis in these patients. Moreover, immune-checkpoint inhibitors such as nivolumab have clinical utility as third-line regimens in patients with unresectable advanced or recurrent gastric cancer [12]. Multimodal treatments, including surgical resection, continue to improve prognosis in patients with advanced gastric cancer.

## Conclusion

We report a long-term survivor of recurrent esophagogastric junction adenocarcinoma, managed with a multimodal treatment that included surgical resection and chemotherapy for metachronous metastases to the lung and upper mediastinal lymph nodes. In the near future, a large-cohort retrospective analysis will be required for assessing the clinical benefits of multimodal treatments.

## Data Availability

Not applicable.

## Abbreviations

CK: Cytokeratin
CT: Computed tomography
FDG-PET: Fluorine-18 fluorodeoxyglucose positron emission tomography
SUV_max_: Maximum standardized uptake value
